# A One-Pot Three-Component Double-Click Method for Synthesis of [^67^Cu]-Labeled Biomolecular Radiotherapeutics

**DOI:** 10.1038/s41598-017-02123-2

**Published:** 2017-05-15

**Authors:** Katsumasa Fujiki, Shinya Yano, Takeshi Ito, Yuki Kumagai, Yoshinori Murakami, Osamu Kamigaito, Hiromitsu Haba, Katsunori Tanaka

**Affiliations:** 1 Biofunctional Synthetic Chemistry Laboratory, RIKEN, 2-1 Hirosawa, Wako, Saitama, 351-0198 Japan; 2 RI Applications Team, RIKEN Nishina Center for Accelerator-Based Science, 2-1 Hirosawa, Wako, Saitama, 351-0198 Japan; 3 The Institute of Medical Science, The University of Tokyo, 4-6-1 Shirokanedai, Minato-ku, Tokyo, 108-8639 Japan; 4 Accelerator Group, RIKEN Nishina Center for Accelerator-Based Science, 2-1 Hirosawa, Wako, Saitama, 351-0198 Japan; 5 Biofunctional Chemistry Laboratory, A. Butlerov Institute of Chemistry, Kazan Federal University, 18 Kremlyovskaya street, Kazan, 420008 Russia; 6 JST-PRESTO, Hirosawa, Wako-shi, Saitama, 351-0198 Japan

## Abstract

A one-pot three-component double-click process for preparing tumor-targeting agents for cancer radiotherapy is described here. By utilizing DOTA (or NOTA) containing tetrazines and the TCO-substituted aldehyde, the two click reactions, the tetrazine ligation (an inverse electron-demand Diels-Alder cycloaddition) and the RIKEN click (a rapid 6π-azaelectrocyclization), could simultaneously proceed under mild conditions to afford covalent attachment of the metal chelator DOTA or NOTA to biomolecules such as to albumin and anti-IGSF4 antibody without altering their activities. Subsequently, radiolabeling of DOTA- or NOTA-attached albumin and anti-IGSF4 antibody (an anti-tumor-targeting antibody) with [^67^Cu], a β^−^-emitting radionuclide, could be achieved in a highly efficient manner via a simple chelation with DOTA proving to be a more superior chelator than NOTA. Our work provides a new and operationally simple method for introducing the [^67^Cu] isotope even in large quantities to biomolecules, thereby representing an important process for preparations of clinically relevant tumor-targeting agents for radiotherapy.

## Introduction

Radiogenic therapies represent an important approach to treatment of cancers. Carbon-ion based radiations^1^ have been one of the most common cancer therapeutic methods. To improve the therapeutic effect of radiations, sensitizers using nano-materials such as gold nanoparticle, magnetic nanoparticles, and quantum dots have been developed recently^2–4^. Unfortunately, such usage have been restricted to treatment of stomach cancer and bowel cancer. More critically, access to radiations targeting specifically to cancer cells remains a huge challenge. On the other hand, radioisotopes (RI) have emerged as power radio-therapeutic agents and have been widely utilized in clinical practices. Radionuclide such as isotope [^89^Sr] has been employed for metastatic bone cancers^5^ and isotope [^131^I] is used as radio-therapeutic medicine for thyroid cancers^6^. More importantly, radiolabeled biomolecules have become more useful as tumor-targeting drugs for specific radiations. For example, the [^90^Y]-labeled anti-CD20 antibody has been developed for clinical usage in the treatment of malignant lymphomas.

Consequently, recent efforts have been devoted to development of radiolabeled tumor-targeting biomolecules, and particularly, in evolving new and efficient synthetic methods for incorporating radionuclides into biomolecules. Some simple and well-known radiolabeling methods would involve assembly of metal chelating moieties and subsequent introduction of a radioisotopic label. More specifically, amidations of lysine residues using activated esters such as succinimidyl ester^7^, or Michael additions of thiols to maleimides^8^ have been made available to attach a metal chelator onto peptides and antibodies. Recently, click chemistry such as Cu(I)-accelerated Huisgen [3 + 2] cycloadditions^9, 10^, strain-promoted [3 + 2] cycloadditions^11^, and inverse electron demand Diels-Alder reactions^12^ have been used for chemoselective and high yielding methods for radiolabelling. However, while selective and efficient introduction of radioactive tags to complex and highly functionalized bioactive molecules could be achieved using click reactions, efficient and regioselective introduction of radiolabels still presents a challenge. In addition, these click methods require key functional groups such as azides, alkynes, tetrazines, and *trans*-alkenes be chemically and/or genetically pre-installed within biomolecules^13–16^. Therefore, a direct radiolabeling via click reactions without overt structural modifications of biomolecules should be more ideal.

In pursuant of such ideal click process, in which the labeling can be performed simply by mixing a native biomolecule with the probe solution under mild conditions, our lab reported a direct reaction of lysine residue on the side chain of peptides via a rapid 6π-azaelectrocyclization (RIKEN click reaction)^17–23^. Fluorescence, positron emitter labels and biofunctional molecules are efficiently and conveniently introduced into the amino groups of the proteins and on the cell surfaces via a reaction involving unsaturated aldehyde probe (such as compound **1** in Fig. 2) at low concentrations over a short period of time at room temperature. Although our RIKEN click method is not bioorthogonal with respect to the natural primary amino groups, the mild reaction conditions yield the preferential and selective labeling of the most exposed and densely expressed amines^24–28^. RIKEN click process hardly proceeds with internal lysines in a tertiary protein or the *N*-terminal amines; however, the lysine residues at the protein surface react rapidly. Bioconjugation therefore occurred preferentially at the surface positions. RIKEN click method thus minimizes indiscriminate amino modification or interference with the native protein functions while introducing new functionalities to solvent-exposed residues. The dihydropyridine electrocyclization products, which preserved the cationic charges of the original lysine residues, contribute to the retention of the native protein activity. This is entirely different from the conventional NHS-ester reaction, which generally proceeds under high reagent concentrations (~10^−2^ M) and long reaction times (a few to several hours), hence indiscriminately modifies the key lysines, resulting in killing native activity of biomolecules.

**Figure 1:**
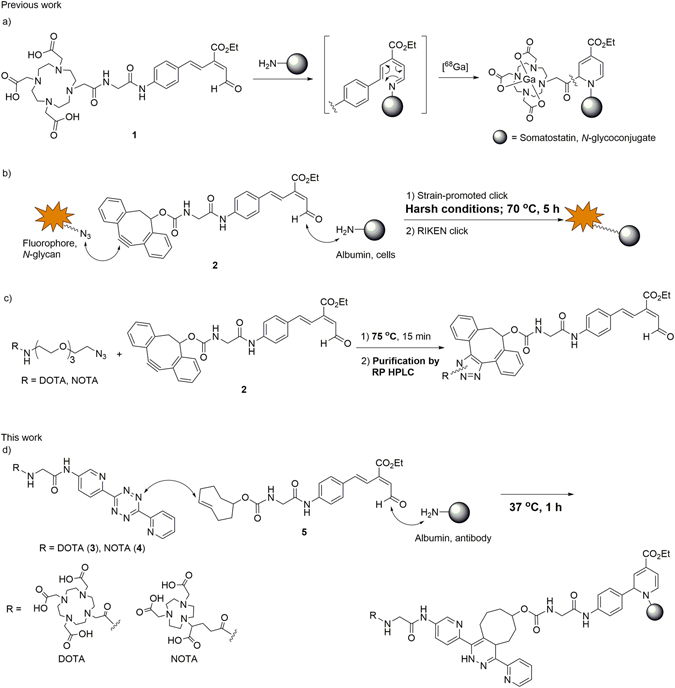
Radiolabeling using the RIKEN click reaction. DOTA: 1,4,7,10-tetraazadodecane-1,4,7,10-tetraacetic acid; NOTA: 1,4,7-triazacyclononane-1,4,7-triacetic acid; TCO: *trans*-cyclooctene.

As examples of RIKEN click reaction applied to biomolecule labeling, we succeeded in preparing the [^68^Ga]-labeled somatostatin and *N*-glycoconjugates via introduction of a DOTA chelating motif (DOTA: 1,4,7,10-tetraazadodecane-1,4,7,10-tetraacetic acid) using the RIKEN click process with DOTA containing aldehyde **1**, followed by chelation with the [^68^Ga] metal radioisotope (Fig. 1a)^17–19^. This radiolabeling enabled *in vivo* visualization of their kinetics for the first time. However, due to the difficulty in synthesizing and handling of **1**, a more general application of RIKEN click reaction for radiolabeling remains elusive.

To develop a facile preparation of the tag-substituted aldehyde, we synthesized aldehyde **2** substituted with a dibenzocyclooctyne (DIBO) motif based on Boons’ report (Fig. 1b)^29^. Strain-promoted click reaction using aldehyde **2** allowed incorporations of reporter groups such as fluorophores or *N*-glycans, and the ultimate introduction into proteins^30–33^ and live cells^34, 35^ through the ensuing RIKEN click reaction. However, heating at 70 °C^30–33^ and/or prolonged reaction time (5 h)^35^ were required for the strain-promoted click reaction. Furthermore, during our preliminary trials of incorporating DOTA, and purification of the click product was also necessary because of low efficiency (Fig. 1c). Thus, to develop a facile and near-quantitative entry to radiolabelled biomolecules, we envisioned DOTA (or NOTA) containing tetrazine **3** (or **4**) and the TCO-substituted aldehyde **5** (NOTA: 1,4,7-triazacyclononane-1,4,7-triacetic acid, tetrazine: 3,6-Di-(2-pyridyl)-*s*-tetrazine, TCO: *trans*-cyclooctene) could be implemented in a one-pot three-component double-click process to radiolabel proteins and antibodies such as albumin and anti-IGSF4 (Immunoglobulin superfamily member 4) (Fig. 1d). We wish to report herein a new and practical method for introducing radiolabels to proteins and antibodies that could serve as tumor-targeting radio-therapeutics.

**Figure 2:**
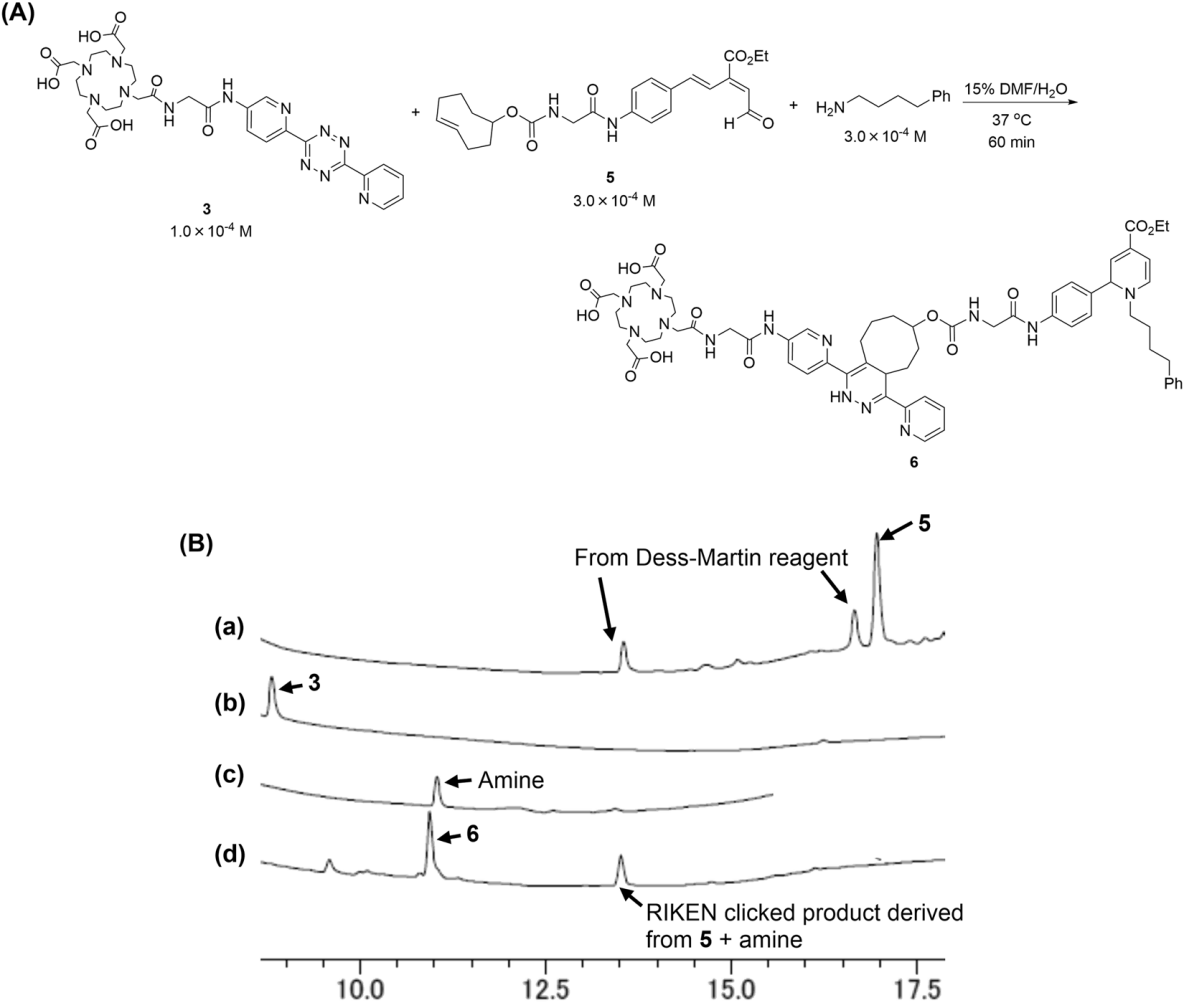
One-pot three-component double-click labeling with 4-phenyl-1-butylamine as a model primary amine. (**A**) Scheme and conditions. (**B**) Reverse phase HPLC analysis. Charts indicate: (a) TCO-substituted aldehyde **5**, (b) DOTA-substituted tetrazine **3**, (c) 4-phenyl-1-butylamine, and (d) reaction mixture. In chart (a), a peak at 16.8 min corresponded to aldehyde **5** and other two peaks at 13.6 and 16.4 min are derived from Dess-Martin reagent during oxidation of the alcohol precursor synthesizing to TCO-aldehyde **5**. In chart (d), two peaks at 10.9 and 13.5 min corresponded to three-component coupling product **6** (m/z 1182.8 calcd for C_60_H_71_N_13_O_13_ [M + H]^+^) and RIKEN clicked product derived from three-fold excess **5** and 4-phenyl-1-butylamine (m/z 584.3 calcd for C_35_H_42_N_3_O_5_ [M − H]^+^). DMF = *N*,*N*-Dimethylformamide.

## Results and Discussion

### One-pot three-component labeling using both the tetrazine ligation and RIKEN click reaction

To identify a more reactive “click” reaction that can be employed in conjunction with our RIKEN click reaction, we were inspired by the tetrazine ligation, which is an inverse electro-demand Diels-Alder reaction that was developed by Fox^36^. We expected that the tetrazine ligation could be complete under mild conditions similar to those for the RIKEN click. More specifically, we thought that both click reactions could be carried out simultaneously in one–pot. Toward this goal, we synthesized DOTA-substituted tetrazine **3** and NOTA-substituted tetrazine **4** as metal chelating motifs, and the TCO-substituted aldehyde **5** as the RIKEN click partner (see Supporting Information).

To evaluate reactivity and compatibility of those click components, 1.0 × 10^−4^ M of the DOTA-substituted tetrazine **3**, 3.0 × 10^−4^ M of the TCO-substituted aldehyde **5**, and 3.0 × 10^−4^ M of 4-phenyl-1-butylamine (serving as a model primary amine of lysines in biomolecules) were reacted by simply mixing them in 15% DMF-containing water and heating at 37 °C (Fig. 2). After 1 h, reverse phase HPLC analysis showed that all of these starting materials were clearly consumed and that the desired double-click product **6** was detected at t = 10.9 min, accompanied by the RIKEN clicked product between excess amount of **5** and amine at t = 13.5 min (Fig. 2d). This result suggests that tetrazine ligation was efficiently completed under comparably mild conditions as those adopted for the RIKEN click reaction, thereby rendering a one-pot three-component coupling labeling feasible.

### One-pot three-component click labeling of albumin and antibody

Labeling of proteins and antibodies without the loss of functions represents one of the major challenges. In our previous report on RIKEN click labeling of DOTA (Fig. 1a), affinity of the DOTA-labeled anti-GFP antibody retained with that of intact antibody^17^. Given that we had some success with the DOTA-attached anti-GFP antibody for the RIKEN click reaction (Fig. 1a), which retained its activity^17^, we investigated our new one-pot three-component double-click reaction on both human serum albumin and anti-IGSF4 as models.

**Figure 3:**
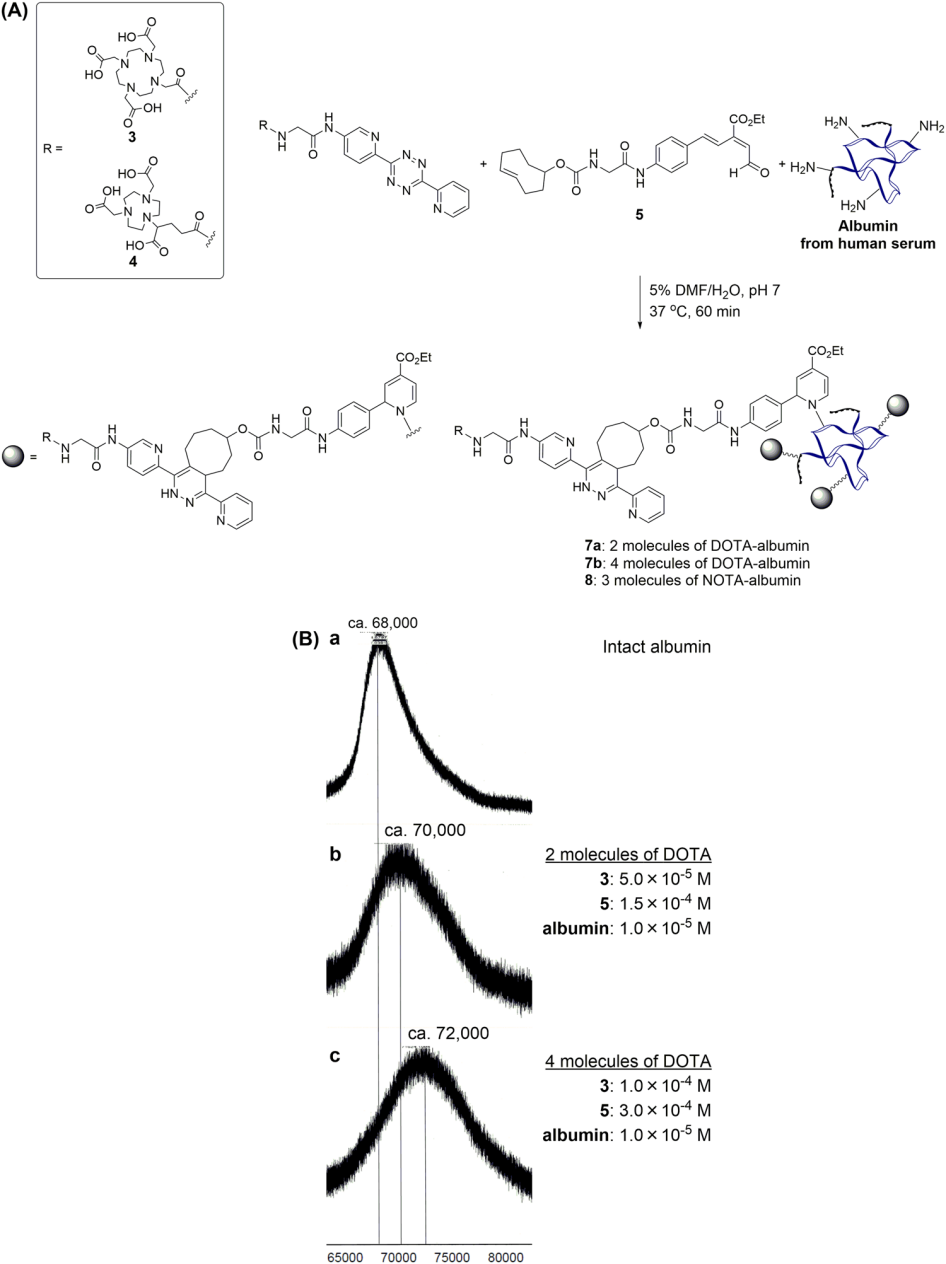
(**A**) One-pot three-component click labeling of albumin using the tetrazine ligation and RIKEN click reaction. (**B**) MALDI-TOF-MS analysis of intact albumin and each product obtained using the one-pot three-component click labeling. Spectra show: (a) Intact albumin. (b) DOTA-labeled albumin performed by using **3** and **5** at concentrations of 5.0 × 10^−5^ M and 1.5 × 10^−4^ M, and (c) 1.0 × 10^−4^ M and 3.0 × 10^−4^ M, for 60 min. DMF = *N*,*N*-Dimethylformamide.

Based on conditions described above, a one-pot three-component double-click reaction was conducted on albumin using the DOTA containing tetrazine **3** or NOTA containing tetrazine **4** and the TCO-substituted aldehyde **5** in 5% DMF-containing aqueous solution at 37 °C (pH 7) for 60 min (Fig. 3A). The resulting product was analyzed by MALDI-TOF-MS and the number of the attached molecules (1,119 of MW increase for 1 molecule of **3** + **5**) was determined by difference of the molecular weight from that of intact albumin. When the one-pot three-component click reaction was carried out with 5.0 × 10^−5^ M of **3**, 1.5 × 10^−4^ M of **5**, and 1.0 × 10^−5^ M of albumin, 2 molecules of DOTA were introduced to albumin (Fig. 3B-b). To further optimize the reaction, when 1.0 × 10^−4^ M of **3** and 3.0 × 10^−4^ M of **5** were treated with albumin, attachment of 4 molecules of DOTA was predominantly observed (Fig. 3B-c). In the case of attaching NOTA to albumin, the one-pot double click reaction under the same conditions as shown in Fig. 3B-c lead to approximately 3 molecules of NOTA-attached albumin were obtained (see Supporting Information). Thus, the number of DOTA motif introduced to albumin by the one-pot three-component procedure could be precisely controlled through adjusting the concentration of the respective click partner. This phenomenon is consistent with those previously reported reactivity of RIKEN click reaction in terms of the efficiency^17^.

**Figure 4:**
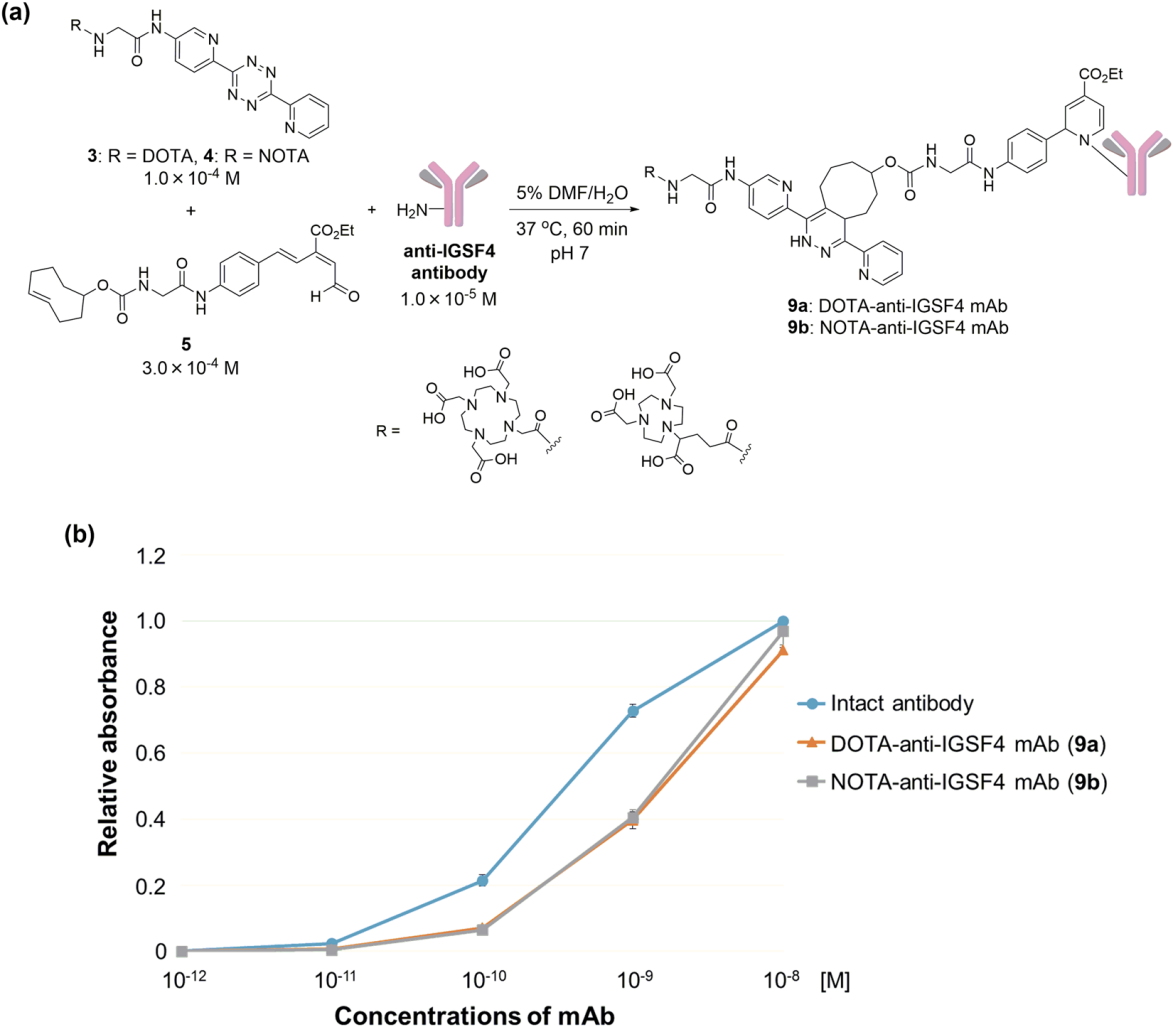
(**a**) One-pot three-component click labeling of anti-IGSF4 antibody as cancer-targeting agent. (**b**) Affinities of intact and labeled anti-IGSF4 antibodies to IGSF4 analyzed by ELISA. DMF = *N*,*N*-Dimethylformamide.

**Figure 5:**
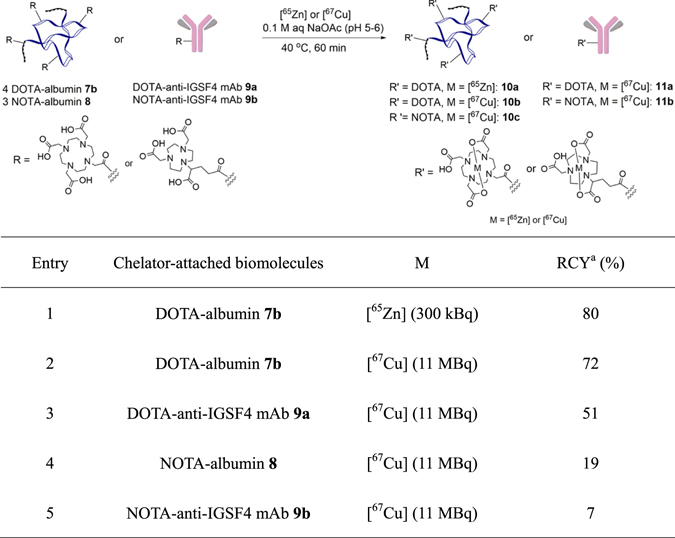
Radiolabelings of DOTA or NOTA-attached albumins and anti-IGSF4 antibody. ^*a*^Radiochemical yield. Specific activity of ^67^Cu and ^65^Zn was 110 MBq μg^−1^ and 125 MBq μg^−1^, respectively.

Encouraged by the success of this one-pot double-click process using albumin, anti-IGSF4 antibody, a potential tumor-targeting agent, was attached with DOTA and NOTA using the conditions established in Fig. 3. Thus, one-pot three-component labeling was performed by treating 1.0 × 10^−4^ M of **3**, 3.0 × 10^−4^ M of **5** and 1.0 × 10^−5^ M of anti-IGSF4 antibody in 5% DMF-containing aqueous solution at 37 °C (pH 7) for 60 min, similar to those applied in Fig. 3B-c (Fig. 4a). Based on the radioactivity of [^67^Cu] incorporated into DOTA-labeled anti-IGSF4 antibody (*vide infra*, see Fig. 5), approximately 3 DOTA molecules were incorporated for each antibody under the conditions. The antigen recognizing activity of DOTA- and NOTA-attached anti-IGSF4 antibodies **9a** and **9b** were measured by enzyme-linked immunosorbent assay (ELISA), and were found to be same as that of the intact anti-IGSF4 antibody (Fig. 4b). Thus, our new one-pot three-component click process was not obstructive to the antibody activity. As previously found for RIKEN click reaction^17^, the labeling might preferentially proceed at sterically non-hindered position of proteins without inhibiting the activity, such as the lysines at Fc moiety of antibody.

### Radiolabeling of DOTA or NOTA-attached albumin and anti-IGSF4 antibody

With DOTA/NOTA-labeled albumin and anti-IGSF4 antibody efficiently and non-invasively prepared via the one-pot three-component click labeling, we pursued radiolabeling of them with [^67^Cu] (a β^−^emitting radionuclide) as an application of labeling of tumor-targeting agents. The use of [^67^Cu] as a RI has received much attention as a highly useful radionuclide for cancer radiotherapy^37^.

The radioisotope of [^67^Cu] was produced in the [^70^Zn(*d*,*αn*)][^67^Cu] reaction (see details in Experimental Section and Supporting Information). The specific radioactivity of [^67^Cu] with 110 MBq μg^−1^ was obtained^38^. [^67^Cu]-Labeling of DOTA- and NOTA-linked albumins and anti-IGSF4 antibodies were then performed with pH being approximately 5–6 using optimized conditions reported by Chen^39^. Prior to labeling with [^67^Cu], radiolabeling of DOTA/NOTA-linked albumin and anti-IGSF4 antibody with [^65^Zn]^40, 41^ was initially evaluated as a model metallic radionuclide (Entry 1 in Fig. 5). When 1.0 × 10^−5^ M of DOTA-linked albumin **7b** (4 DOTA are labeled, see Fig. 3B-c) was treated with 300 kBq of [^65^Zn] in aqueous sodium acetate at 40 °C for 1 h, the chelation to the DOTA motif was successful with a radiochemical yield (RCY) of 80% (**10a**, Entry 1). With this success in hand, labeling with [^67^Cu] was performed. As shown in Entry 2, treatment of DOTA-linked albumin **7b** with 11 MBq of [^67^Cu] afforded [^67^Cu]-labeled albumin **10b** in 72% RCY. [^67^Cu]-Labeling of DOTA-linked anti-IGSF4 antibody **9a** was similarly successful to afford [^67^Cu]-labeled anti-IGSF4 antibody **11a** in a practical RCY of 51% (Entry 3). To probe the selective chelation of these radioactive metals to DOTA-linked albumin or anti-IGSF4 antibody, albumin and anti-IGSF4 antibody without the DOTA motif were subjected to the same reaction conditions. Neither [^65^Zn] nor [^67^Cu] was found to be incorporated, thereby suggesting that the labeling of these metal radionuclides could occur only with albumin or antibody linked to the DOTA chelator (see Supporting Information). Given that chelation of Cu^2+^ metal ion is dependent upon the amount of DOTA linked to antibodies, it could be estimated that approximately 3 molecules of DOTA were found introduced onto antibodies based on RCY of 72% and 51% for [^67^Cu]-labeled DOTA-linked albumin **10b** and labeled antibody **11a**, respectively (See also labeling analysis in Fig. 4).

Lastly, [^67^Cu]-labelings of NOTA-linked albumin and anti-IGSF4 antibody **8** and **9b** were evaluated under same conditions employed for DOTA-attached systems. Unfortunately, [^67^Cu]-labeled NOTA-albumin **10c** and anti-IGSF4 antibody **11b** were obtained in just 19% and 7% of RCY (Entries 4 and 5), which are much lower those of DOTA-attached systems **7b** and **9a**. The low efficiency in the metal chelation is likely due to the structural rigidity of the NOTA chelator^42^ that usually require a much high temperature and a longer incubation time^43^. Our studies suggest that DOTA is a more suitable metal chelator than NOTA for radiolabeling of unstable biomolecules such as antibodies at low temperatures.

## Conclusion

In summary, we have developed a one-pot three-component double-click process for preparing tumor-targeting agents for cancer radiotherapy. Specifically, by employing DOTA- or NOTA containing tetrazines and the TCO-substituted aldehyde, the two click reactions, the tetrazine ligation and the RIKEN click reaction, could simultaneously take place to covalently attach DOTA or NOTA to biomolecules without disrupting their activities or destabilizing them. Subsequently, concise preparations of [^67^Cu]-labeled albumin and anti-IGSF4 antibody (anti-tumor-targeting antibody) could be achieved with DOTA being a more superior chelator to the Cu^2+^ metal than NOTA. Our work provides a novel and practical method for introducing the [^67^Cu] metal isotope, a β^−^-emitting radionuclide, even in large quantities to biomolecules that can serve as new cancer radio-therapeutics. Efforts are underway to explore potential clinical applications of this efficient access to tumor-targeting agents.

## Methods

All other commercially available reagents were used without further purification. Distilled water was purchased from nacalai teshque. DMF was purchased from Wako Pure Chemicals Industries Ltd. Syntheses of DOTA-tetrazine **3**, NOTA-tetrazine **4**, and TCO-aldehyde **5** were described in Supporting Information. DMF = *N*,*N*-dimethylformamide, PBS = phosphate buffer saline, HEK293 cells = human embryonic kidney 293 cells.

### One-pot three-component double-click reaction for attaching DOTA to albumin (7a)

Aqueous solution of albumin (1 × 10^−4^ M, 20 μL) was diluted with distilled water (170 μL), then aqueous solution of DOTA-tetrazine **3** (2 × 10^−3^ M, 5 μL) and TCO-aldehyde **5** in DMF (6 × 10^−3^ M, 5 μL) were added and the mixture was heated to 37 °C. After 1 h, the reaction mixture was transferred into Amicon^®^ 10 K, centrifuge with 14k rpm was performed for 12 min. To the filter was added 10% DMF-containing water (100 μL) and centrifuge with 14k rpm was done for 12 min. Then, to the filter was added distilled water (300 μL) and centrifuge with 14k rpm was done for 12 min. This wash was repeated more than 2 times. The residue on filter was collected and diluted with water to give 2 × 10^−5^ M of **7a** in water as stock solution.

### One-pot three-component doubl-click reaction for attaching DOTA to albumin (7b)

According to the procedure of one-pot three-component double click labeling for preparation of **7a**, the labeling was carried out using distilled water (160 μL), aqueous solution of albumin (1 × 10^−4^ M, 20 μL), aqueous solution of the DOTA containing tetrazine **3** (2 × 10^−3^ M, 10 μL) and TCO-substituted aldehyde **5** in DMF (6 × 10^−3^ M, 10 μL). The stock solution of **7b** (2 × 10^−5^ M) in water was prepared for the subsequent radiolabeling.

### One-pot three-component click reaction for attaching DOTA to anti-IGSF4 antibody (9a)

According to the protocol of one-pot three-component double click labeling of albumin with DOTA (preparation of **7b**), the labeling of anti-IGSF4 antibody with DOTA was carried out. The anti-IGSF4 mouse monoclonal antibody was raised against the extracellular domain of IGSF4 produced by HEK293 cells (Health Science Research Resources Bank, Osaka, Japan). Anti-IGSF4 antibody in PBS (3 × 10^−5^ M) was centrifuged with Amicon^®^ with 14k rpm for 12 min and diluted with distilled water to give anti-IGSF4 antibody in water (2 × 10^−5^ M) prior to use. The labeling was performed with the antibody in water. The DOTA-attached anti-IGSF4 antibody **9a** in water (2 × 10^−5^ M for each) were stocked for radiolabeling.

### One-pot three-component click reaction for attaching NOTA to anti-IGSF4 antibody (9b)

According to the same protocol of one-pot three-component double click labeling of anti-IGSF4 antibody with DOTA (preparation of **9a**), the labeling of anti-IGSF4 antibody with NOTA was carried out. The labeling was performed with the antibody in water. The NOTA-attached anti-IGSF4 antibody **9b** in water (4 × 10^−6^ M for each) were stocked for radiolabeling.

### [^67^Cu] production

The radioisotope of [^67^Cu] was produced in the [^70^Zn(*d*,*αn*)][^67^Cu] reaction. A schematic of the [^67^Cu] production chamber is shown in Supporting Information. The 24-MeV deuteron beam was extracted from the RIKEN AVF cyclotron, and the beam intensity was 4.0 μA. Zinc-70-enriched oxide powder ([^70^Zn]O) was pressed for 3 min at 2.0 × 10^3^ kg cm^−2^ to form a disk of 10-mm diameter and 3.4 × 10^2^ mg cm^−2^ thickness. The isotopic composition of the [^70^Zn]O target was 96.87% [^70^Zn], 1.55% [^68^Zn], 0.09% [^67^Zn], 0.55% [^66^Zn], and 0.94% [^64^Zn]. The [^70^Zn]O disk target was placed on a tantalum beam stopper and covered with a 10-μm aluminum foil (see figures in Supporting Information). During the irradiation, the [^70^Zn]O target was cooled with circulating helium gas (30 L/min) and water (1.5 L/min) at the upstream (aluminum cover) and downstream (tantalum plate) of the beam, respectively. The beam axis was continuously rotated in 3-mm diameter at 2 Hz to avoid a local heating of the [^70^Zn]O target using a beam wobbling electromagnet on the beam line of the AVF cyclotron. After the irradiation for 10 h, [^67^Cu] was separated from the target material and by-product radioisotopes such as [^67^Ga], [^69m^Zn] and [^71^Zn] through the two-step chromatographic separation with the Eichrom Cu resin and the Dowex 1X8 anion-exchange resin.^[16]^ 4 MBq of [^67^Cu] was finally prepared in 300 μL of 0.1 M CH_3_COOH. The radionuclidic purity of the [^67^Cu] solution was evaluated to be >99.9% by γ-ray spectrometry with a germanium semiconductor detector (ORTEC GEM-25185-P). A typical γ-ray spectrum of the purified [^67^Cu] used for radiolabeling is provided in Supporting Information. The chemical purity of the solution was evaluated with an inductively coupled plasma mass spectrometer (Agilent Technologies 7700x). Among the elements having atomic number *Z* ≥ 20, Cu (2.1 ppm) and Br (1.0 ppm) were only detected with concentrations of >1 ppm. The specific radioactivity of [^67^Cu] was then 110 MBq μg^−1^.

### Radiolabeling of DOTA-attached albumin (7b) with [^67^Cu]

To the stock solution of **7b** (2 × 10^−5^ M, 20 μL) was added [^67^Cu] (11 MBq) in 0.1 M of aqueous sodium acetate (pH 5–6, 20 μL) and the mixture was heated at 40 °C for 1 h. The reaction mixture was transferred into Amicon^®^ 10 K and 0.1 M of aqueous acetic acid (pH 5–6) was fully added. Centrifuge was performed with 14k rpm for 12 min. 0.1 M of aqueous acetic acid (pH 5–6, 450 μL) was added and centrifuge was done with 14k rpm for 12 min. This wash was repeated one more time. γ-Ray doses of the residue and filtrate were measured using germanium semiconductor detector.

### ELISA to test affinity of DOTA/NOTA-linked anti-IGSF4 antibodies (9a/b)

The wells of ELISA plate (Nunc-Immuno Plate, Thermo Fisher Scientific, Waltham, MA, USA) were coated with 100 ng/mL IGSF4 protein in coating buffer (50 mM carbonate buffer, pH 9.6) and incubated overnight at 4 °C. After two washes with washing buffer (phosphate buffered saline (PBS) containing 0.05% (v/v) Tween-20), the wells were blocked with 1% BSA in PBS for 1 h at RT. Anti-IGSF4 antibodies conjugated with DOTA and NOTA were serially diluted with PBS, added to the wells, and incubated for 1 h at RT. After four washes with buffer, horseradish peroxidase (HRP)-conjugated goat anti-mouse IgG antibody (Merck Millipore, Darmstadt, Germany) in PBS was added to the wells and incubated for 1 h at RT. After six washes with washing buffer, color development was performed by incubation with TMB solution (ScyTek, Logan, UT, USA) for 10 min at RT and was stopped by addition of TMB Stop Buffer (ScyTek). Finally, the absorbance of 450 nm was detected using a microplate reader (PerkinElmer, Waltham, MA, USA).

## Electronic supplementary material

Supplementary Information
