# Supplementary material for: A One-Pot Three-Component Double-Click Method for Synthesis of [67Cu]-Labeled Biomolecular Radiotherapeutics
Source: Sci Rep. 2017 May 15;7:1912. doi: 10.1038/s41598-017-02123-2 (PMC5432496; doi:10.1038/s41598-017-02123-2)
Supplement: Supplementary file 1 — Supplementary Information [file 41598_2017_2123_MOESM1_ESM.pdf]

Supplementary Information

A One-Pot Three-Component Double-Click Method for Synthesis of [^{67}Cu]-Labeled Biomolecular Radiotherapeutics

Katsumasa Fujiki, Shinya Yano, Takeshi Ito, Yuki Kumagai, Yoshinori Murakami, Osamu Kamigaito, Hiromitsu Haba, and Katsunori Tanaka*

1. General methods 2

2. Synthesis of DOTA-3,6-di(2-pyridyl)-*s*-tetrazine, (3) 3

3. Synthesis of NOTA-3,6-di(2-pyridyl)-*s*-tetrazine, (4) 5

4. Synthesis of trans-cyclooctene-aldehyde, (5) 6

5. One-pot three-component click labeling of albumin with NOTA, (8) 8

6. A schematic of the [^{67}Cu] production chamber and the spectrum of the produced [^{67}Cu] 9

7. Radiolabeling of DOTA/NOTA-attached albumins and anti-IGSF4 antibodies 10

7.1 [^{65}Zn]labeling of DOTA-albumin (**7b**) 10

7.2 [^{67}Cu]labeling of DOTA/NOTA-albumins (**7b**/**8**) 11

7.3 [^{67}Cu]labeling of DOTA/NOTA-anti-IGSF4 antibodies (**9a**/**9b**) 12

8. NMR spectra 13

8.1 3-{3-[2-(*tert*-butoxycarbonylamino)acetylamino](pyrid-6-yl)}-6-(pyrid-2-yl)-*s*-tetrazine, (**A**) 13

8.2 DOTA-3,6-di(2-pyridyl)-*s*-tetrazine, (**3**) 14

8.3 NOTA-3,6-di(2-pyridyl)-*s*-tetrazine, (**4**) 15

8.4 *trans*-cyclooctene-alcohol, (**C**) 16

8.5 *trans*-cyclooctene-aldehyde, (**5**) 17

9. References 18

1. General methods

All commercially available reagents were used without further purification. All anhydrous solvents were purchased from Wako Pure Chemical Industries Ltd., and TCO succinimidyl ester (TCO-NHS) was purchased from Santa Cruz Biotechnology. *N*-Boc-Gly-OH, HOBt and HATU were purchased from Peptide Institute, Inc. DOTA succinimidyl ester, HPF_6·TFA salt (DOTA-NHS-ester) was purchased from Macrocyclics. NOTA succinimidyl ester (NODA-GA-NHS) was purchased from CheMatech. Other commercially available reagents were purchased from Sigma-Aldrich. Silica gel (Silica gel 60, 0.015–0.040 mm) for column chromatography was used. For preparative TLC, PLC glass plate Silica gel 60 F_{254}, 0.5 or 1 mm (Merck-Millipore) was used. HPLC grade CH_3CN from Sigma-Aldrich was used for HPLC analysis.

Reverse phase HPLC analysis was performed on a Shimadzu Prominence® system equipped with a Develosil™ (NOMURA CHEMICAL Co., Ltd.) (ODS-HG-3, 4.6 x 250 mm). The mobile phase for HPLC were distilled H_2O with 0.1% TFA (Buffer A) and HPLC grade MeCN with 0.1% TFA (Buffer B). High-resolution mass spectra (HRMS) were obtained on a Bruker micrOTOF-QIII spectrometer® by electron spray ionization (ESI-TOF-MS). Mass spectra of DOTA/NOTA-attached albumins **7a**,**b** were obtained on a Bruker autoflex spectrometer® by matrix assisted laser desorption ionization (MALDI-TOF MS), using 2,5-dihydroxybenzoic acid (DHB) as a matrix.

2. Synthesis of DOTA-3,6-di(2-pyridyl)-*s*-tetrazine, (3)

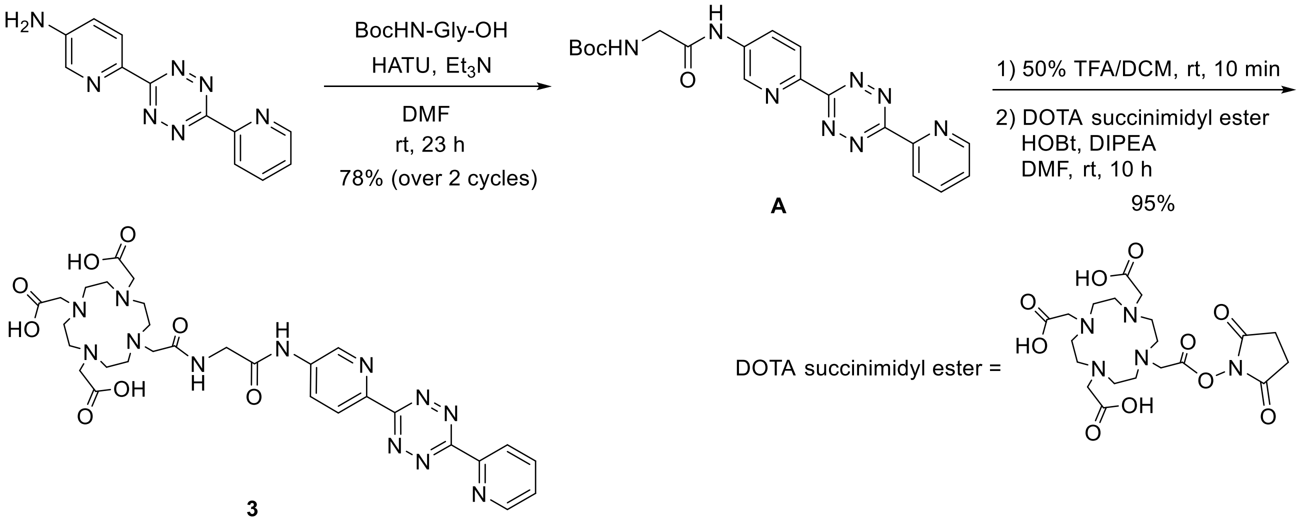

Synthesis of 3-{3-[2-(*tert*-butoxycarbonylamino)acetylamino](pyrid-6-yl)}-6-(pyrid-2-yl)-*s*-tetrazine, (A)

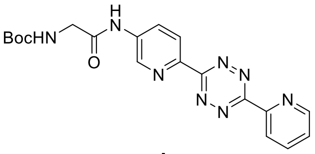

A

3-[(3-amino)pyrid-6-yl]-6-(pyrid-2-yl)-*s*-tetrazine (10.8 mg, 0.043 mmol) synthesized according to the literature,[1] *N*-Boc-Gly-OH (11.2 mg, 0.064), HATU (24.5 mg, 0.064 mmol) were added in oven-dried flask filled with N_2 gas and dissolved in dry DMF (0.29 ml). To the solution was added Et_3N (15.1 μL, 0.11 mmol) and stirred for 23 h at room temperature. The reaction mixture was quenched with brine, extracted with $CHCl_3$. The combined organic layer was concentrated in vacuo, and the obtained crude material was purified by preparative TLC ($CHCl_3$:MeOH 8:1) to give the product (6.5 mg, 37%) as a red solid. The recovery of 3-[(3-amino)pyrid-6-yl]-6-(pyrid-2-yl)-*s*-tetrazine (4.0 mg, 37%) was reused for second cycle, giving the product (2.7 mg, 41%). ^{1}H NMR (300 MHz, $CDCl_3$:CD_3OD 3:1): δ 8.96-8.94 (m, 1H), 8.87 (s, 1H), 8.75 (d, J = 8.4 Hz, 2H), 8.63-8.60 (m, 1H), 8.09-8.02 (m, 1H), 7.65-7.61 (m, 1H), 5.91-5.89 (m, 1H), 4.01 (s, 2H), 1.48 (s, 9H); ^{13}C NMR (75 MHz, $CDCl_3$:CD_3OD 10:1): δ 163.3, 163.0, 150.6, 149.6, 143.9, 141.6, 138.1, 137.7, 127.1, 126.7, 125.1, 124.3, 28.1; HRMS (ESI+) $[M+H]^+$ calcd for $C_{19}H_{21}N_8O_3$ 409.1731, found 409.1743

Synthesis of DOTA-Gly-tetrazine, (3)

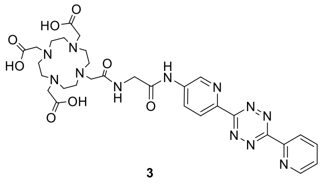

3

A (2.9 mg, 0.0071 mmol) was dissolved in 50% TFA-containing DCM (0.20 mL), stirring for 10 min at room temperature. The reaction mixture was co-evaporated with toluene (3×). Then 1 N HCl in diethyl ether was added and co-evaporated with toluene. To the obtained material were added DOTA succinimidyl ester, HPF_6·TFA salt (5.9 mg, 0.0077 mmol), HOBt (1.4 mg, 0.010 mmol), and DIPEA (7.3 μL, 0.043 mmol), filled with N_2 gas, dissolved in dry DMF (0.14 ml). After stirring for 23 h at room temperature, the reaction was directly transferred onto cosmosil 75C_{18}-OPN (2-propanol to 2-propanol:H_2O 5:95) to afford the product (4.7 mg, 95%) as red solid. ^1H NMR (400 MHz, D_2O): δ 8.72-8.69 (m, 1H), 8.63 (s, 1H), 8.44-8.41 (m, 2H), 8.14-8.09 (m, 1H), 8.01-7.99 (m, 1H), 7.61-7.57 (m, 1H), 4.00 (s, 2H), 3.74 (s, 4H), 3.61-3.44 (m, 3H), 3.38-3.31 (m, 7H), 3.15 (s, 2H), 3.01-2.93 (m, 8H); HRMS (ESI−) $[M-H]^-$ calcd for $C_{30}H_{37}N_{12}O_8$ 693.2863, found 693.2847

3. Synthesis of NOTA-3,6-di(2-pyridyl)-*s*-tetrazine, (4)

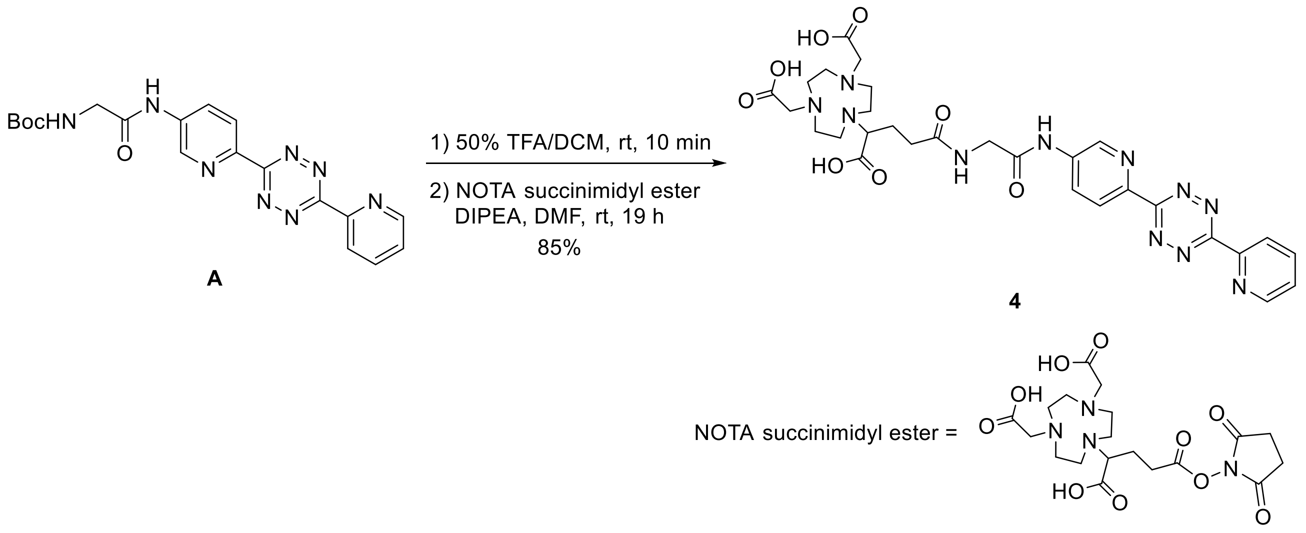

According to the protocol of synthesis of **3**, **4** was preprared from *N*-Boc-Gly-tetrazine (1.1 mg, 0.0027 mmol) and NOTA succinimidyl ester, HPF_6·TFA salt (1.8 mg, 0.0025 mmol). Purification by cosmosil $75C_{18}$-OPN (MeCN to MeCN:H_2O 10:90) to give the product (1.4 mg, 85%) as red solid. ^1H NMR (400 MHz, D_2O): δ 8.84-8.82 (m, 1H), 8.74-8.73 (m, 1H), 8.60 (d, *J* = 7.6 Hz, 2H), 8.22 (dd, *J* = 7.2, 1.6 Hz, 1H), 8.11 (dd, *J* = 7.2, 1.6 Hz, 1H), 7.69-7.68 (m, 1H), 4.04-4.03 (m, 1H), 3.64 (brs, 4H), 3.51-3.42 (m, 2H), 3.15-3.02 (m, 10H), 2.04-2.77 (m, 2H), 2.48-2.30 (m, 2H), 2.09-1.92 (m, 2H); HRMS (ESI+) $[M+Na]^+$ calcd for $C_{29}H_{35}N_{11}O_8Na$ 688.2563, found 688.2562

4. Synthesis of *trans*-cyclooctene (TCO)-aldehyde, (5)

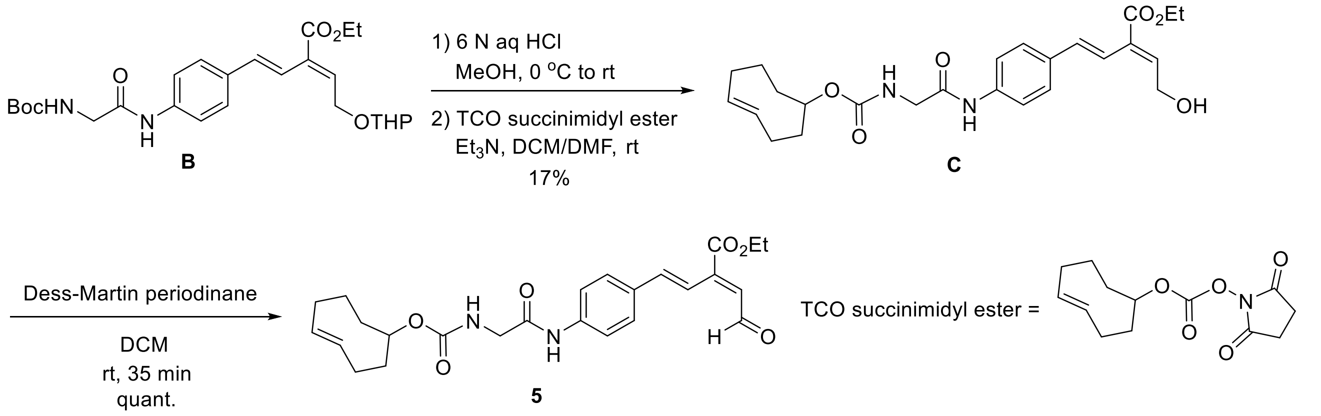

Synthesis of *trans*-cyclooctene (TCO)-alcohol, (C)

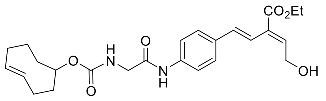

C

To the solution of *N*-Boc-Gly-aldehyde (**B**) (21 mg, 0.043 mmol) in MeOH (0.29 mL) was added 6 N aqueous HCl (0.29 mL) at 0 °C, then warmed to room temperature, stirred for 2 h. To the reaction mixture were added ethyl acetate and 0.5 M of aqueous HCl, and the aqueous layer was concentrated under reduced pressure, giving the Boc/THP-deprotected product hydrochloride as yellow oil. The deprotected product (8.7 mg, 0.026 mmol) in the flask filled with N_2 gas was dissolved in dry DMC (0.14 mL) and dry DMF (0.070 mL). To the solution were added TCO succinimidyl ester (5.2 mg, 0.020 mmol), Et_3N (6.7 μL, 0.048 mmol) and the reaction mixture was stirred for 3.5 h at room temperature. The reaction was quenched with 0.5 N aqueous HCl and extracted with $CHCl_3$. The combined organic layer was washed with sat. aqueous $NaHCO_3$, brine, dried over Na_2SO_4, and filtered. The filtrate was concentrated under reduced pressure, the crude product was purified by preparative TLC ($CHCl_3$:MeOH 10:1) to give the product (3.1 mg, 34%) as very light yellow solid. ^{1}H NMR (400 MHz, $CDCl_3$): δ 8.11 (brs, 1H), 7.49 (d, *J* = 8.4 Hz, 2H), 7.39 (d, *J* = 8.4 Hz, 2H), 6.84-6.78 (m, 3H), 5.58-5.50 (m, 2H), 5.29 (brs, 1H), 4.57 (d, *J* = 6.4 Hz, 2H), 4.43-4.40 (m, 1H), 4.28 (q, *J* = 6.8 Hz, 2H), 3.95 (d, *J* = 5.6 Hz, 2H), 2.40-2.32 (m, 4H), 2.03-1.92 (m, 4H), 1.77-1.72 (m, 2H), 1.35 (t, *J* = 6.8 Hz, 3H); HRMS (ESI+) $[M+Na]^+$ calcd for $C_{25}H_{32}N_2O_6Na$ 479.2173, found 479.2153

Synthesis of *trans*-cyclooctene (TCO)-aldehyde, (5)

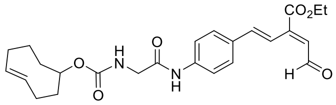

5

TCO-alcohol **5** (0.2 mg, 0.004 mmol) and Dess-Martin reagent (0.4 mg, 0.94 μmol) was dissolved in dry DCM (0.24 mL), stirred for 35 min at room temperature. The reaction mixture was filtered and the filtrate was concentrated under reduced pressure to give the product as yellow solid. The product was

used immediately for one-pot three-component double click labeling. ^{1}H NMR (400 MHz, $CDCl_3$): δ 10.15 (d, *J* = 7.6 Hz, 1H), 8.18 (brs, 1H), 7.57-7.45 (m, 4H), 7.34 (d, *J* = 16.4 Hz, 1H), 7.06 (d, *J* = 16.4 Hz, 1H), 6.66 (d, *J* = 7.2 Hz, 1H), 5.58-5.51 (m, 2H), 5.21 (brs, 1H), 4.36 (q, *J* = 7.2 Hz, 2H), 3.96-3.95 (m, 3H), 2.39-2.36 (m, 4H), 2.05-1.93 (m, 4H), 1.55-1.36 (m, 5H); HRMS (ESI+) $[M+Na]^+$ calcd for $C_{25}H_{30}N_2O_6Na$ 477.1996, found 477.2013.

5. One-pot three-component click labeling of albumin with NOTA, (8)

Accoridng to the procedure of one-pot three-component double click labeling for preparation of **7b**, the labeling was carried out by using distilled water (160 μL), aqueous solution of albumin (2.0×10^{-4} M, 20 μL), aqueous solution of NOTA-tetrazine **4** (2.0×10^{-3} M, 10 μL) and TCO-aldehyde **5** in DMF (6.0×10^{-3} M, 10 μL). The stock solution of **8** (2.0×10^{-5} M) in water was prepared for the successive radiolabeling. MALDI-TOF-MASS (positive) detected m/z 72.3 kDa corresponding to the 3 NOTA-attached albumin **8**.

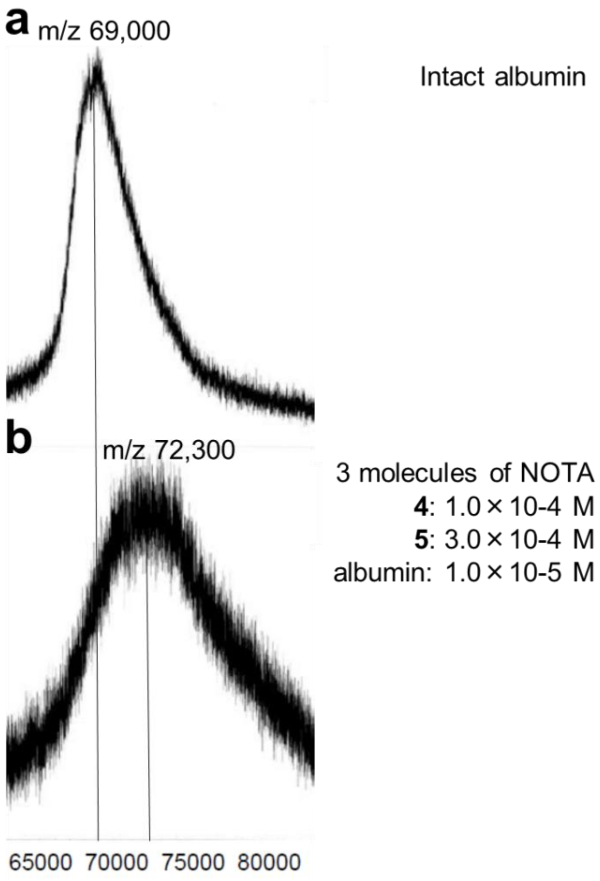

6. A schematic of the [^{67}Cu] production chamber and the spectrum of the produced [^{67}Cu]

(a) A schematic and (b) a photo of the ^{67}Cu production chamber. A [^{70}Zn]O target is irradiated with the 24-MeV deuteron beam from the AVF cyclotron. The target was continuously cooled with circulating water and helium gas. The beam axis is rotated in 3-mm diameter at 2 Hz. (c) A [^{70}Zn]O disk target on a tantalum beam stopper and covered with a 10-μm aluminum foil. (d) A typical γ-ray spectrum of the purified [^{67}Cu] from the enriched [^{70}Zn] target.

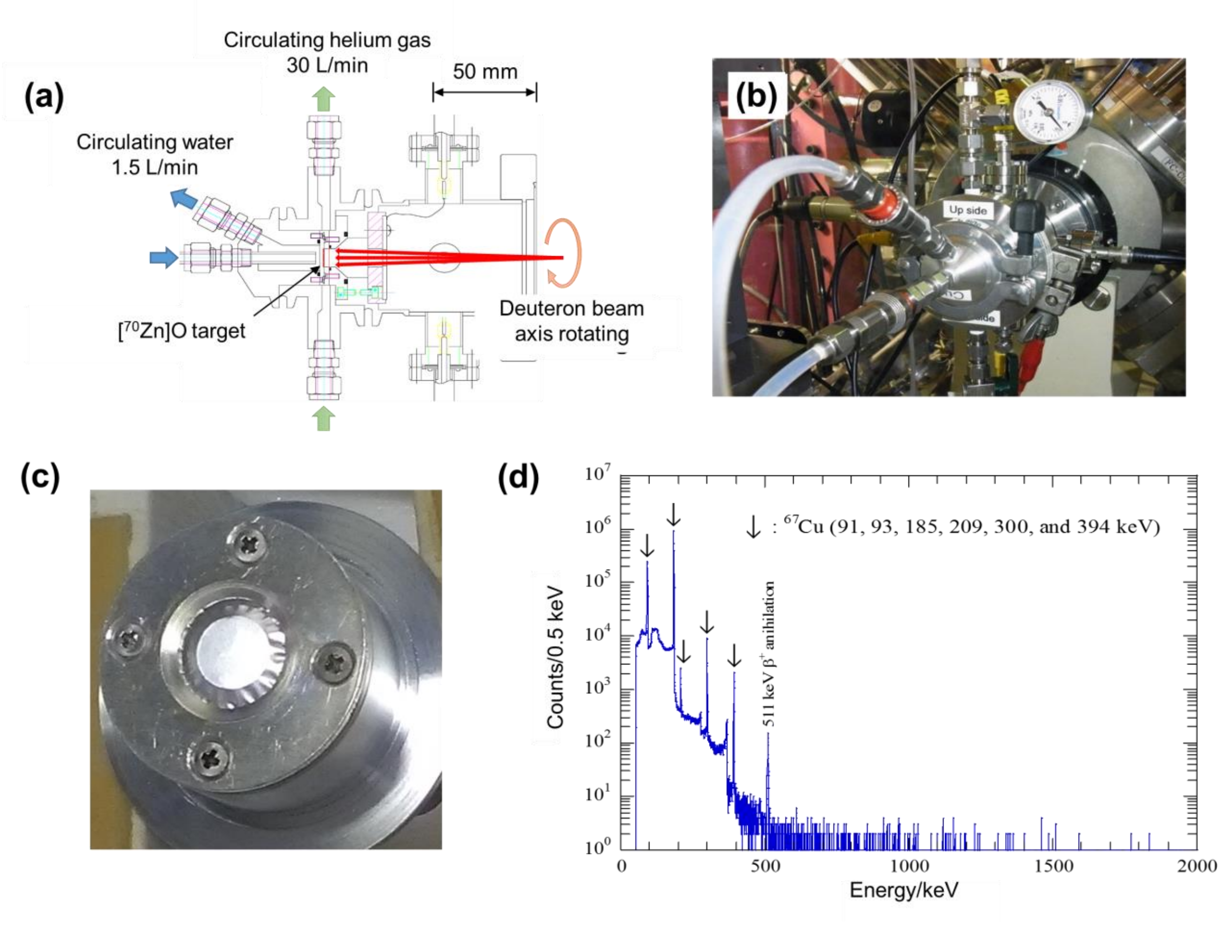

7. Radiolabeling of DOTA/NOTA-albumins and anti-IGSF4 antibodies

7.1 [^{65}Zn]labeling of 4 DOTA-albumin (7b)

According to [^{67}Cu]labeling of **7b**, [^{65}Zn]labeling of **7b** was carried out by using the stock solution of **7b** (2.0×10^{-5} M, 20 µL), [^{65}Zn] (300 kBq) in 0.1 M of aqueous sodium acetate (pH 5−6, 20 µL). The labeling was performed at 40 °C for 1 hour. After the purification by centrifuge with Amicon® 10 K (14 k, 12 min) (2×), the γ-ray dose of the obtained labeled albumin was measured by germanium semiconductor detector.

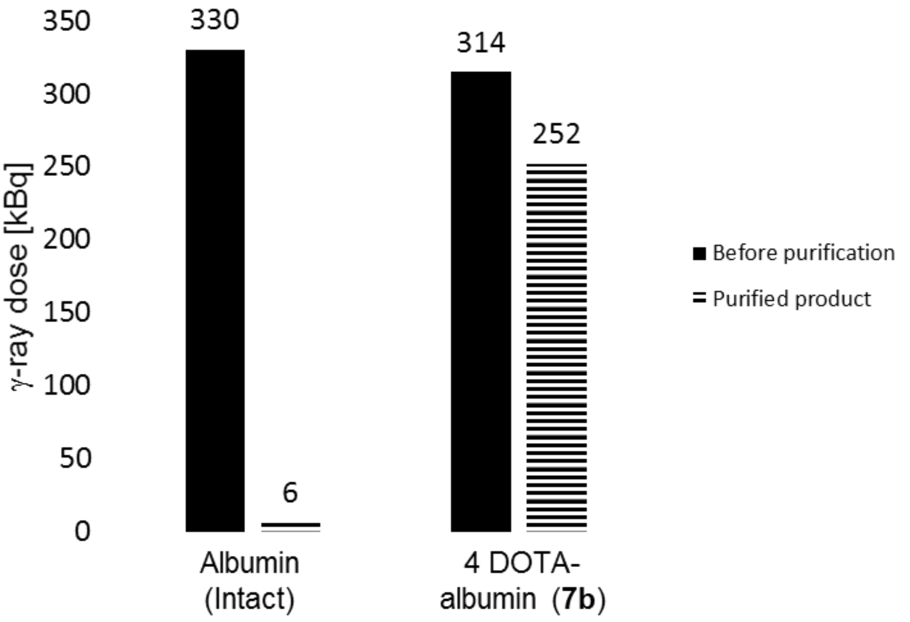

7.2 [^{67}Cu]labeling of 4 DOTA/3 NOTA-albumins (7b/8)

According to [^{67}Cu]labeling of **7b**, [^{67}Cu]labeling of **8** was carried out by using the stock solution of **8** (2.0×10^{-5} M, 20 μL), [^{67}Cu] (11 MBq) in 0.1 M of aqueous sodium acetate (pH 5−6, 20 μL). The labeling was performed at 40 ^{o}C for 1 h. After the purification by centrifuge with Amicon® 10 K (14 k, 12 min) (2×), the γ-ray dose of the obtained labeled albumin was measured by germanium semiconductor detector.

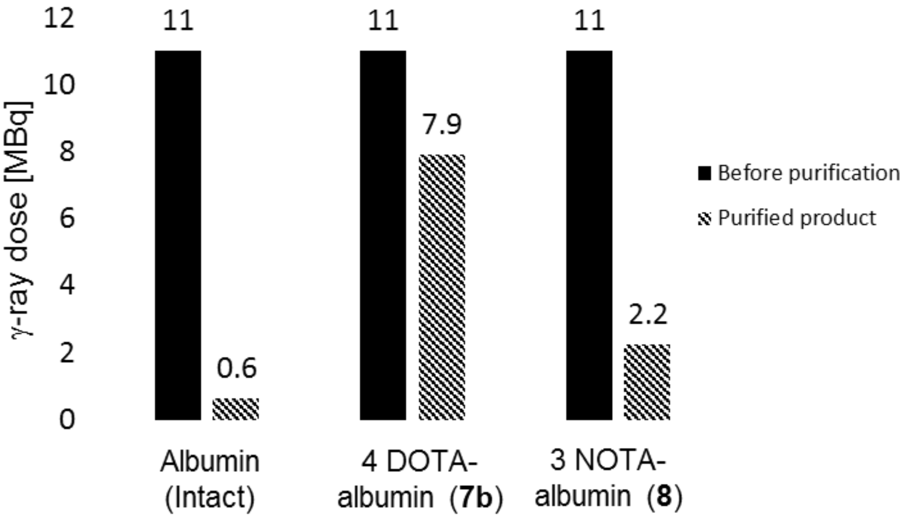

7.3 [^{67}Cu]labeling of DOTA/NOTA-anti-IGSF4 mAb (9a/9b)

According to [^{67}Cu]labeling of **7b**, [^{67}Cu]labeling of DOTA/NOTA-anti-IGSF4 mAb **9a**/**9b** was carried out by using the stock solution of **9a** (2.0×10^{-5} M, 20 µL) or **9b** (4.0×10^{-6} M, 20 µL), [^{67}Cu] (11 MBq) in 0.1 M of aqueous acetic acid (pH 5−6, 20 µL). The labeling was performed at 40 °C for 1 h. After the purification by centrifuge with Amicon® 10 K (14 k, 12 min) (2×), the γ-ray dose of the obtained labeled albumin was measured by germanium semiconductor detector.

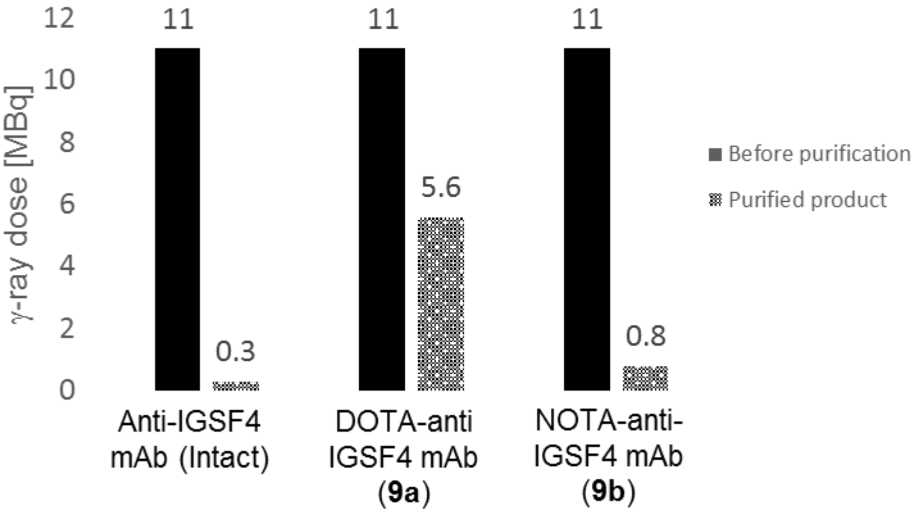

8. NMR spectra

3-{3-[2-(*tert*-butoxycarbonylamino)acetylamino](pyrid-6-yl)}-6-(pyrid-2-yl)-*s*-tetrazine, (**A**)

^{1}H NMR (300 MHz, $CDCl_3$:CD_3OD 3:1)

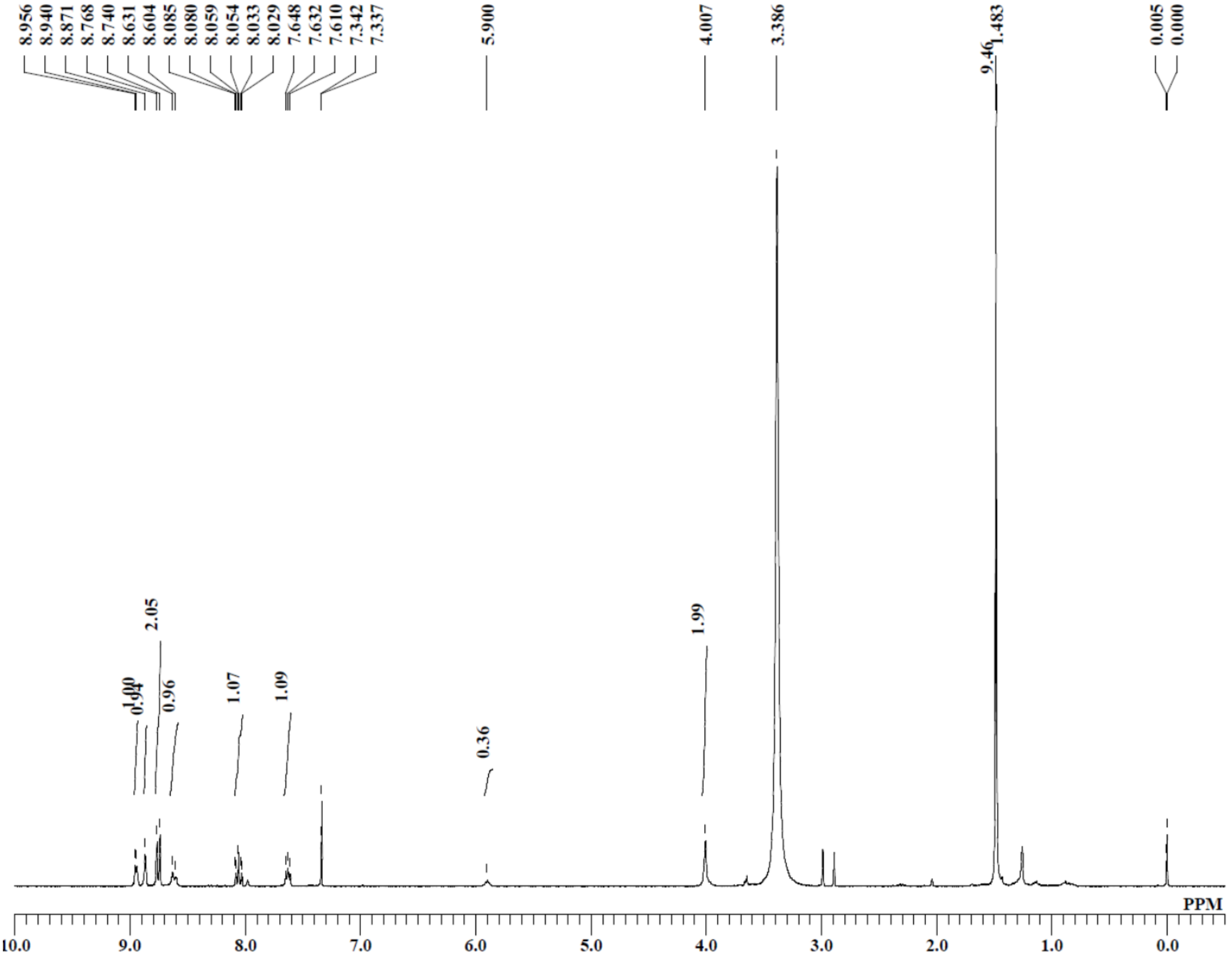

DOTA-3,6-di(2-pyridyl)-*s*-tetrazine, (**3**)

^{1}H NMR (400 MHz, D_2O)

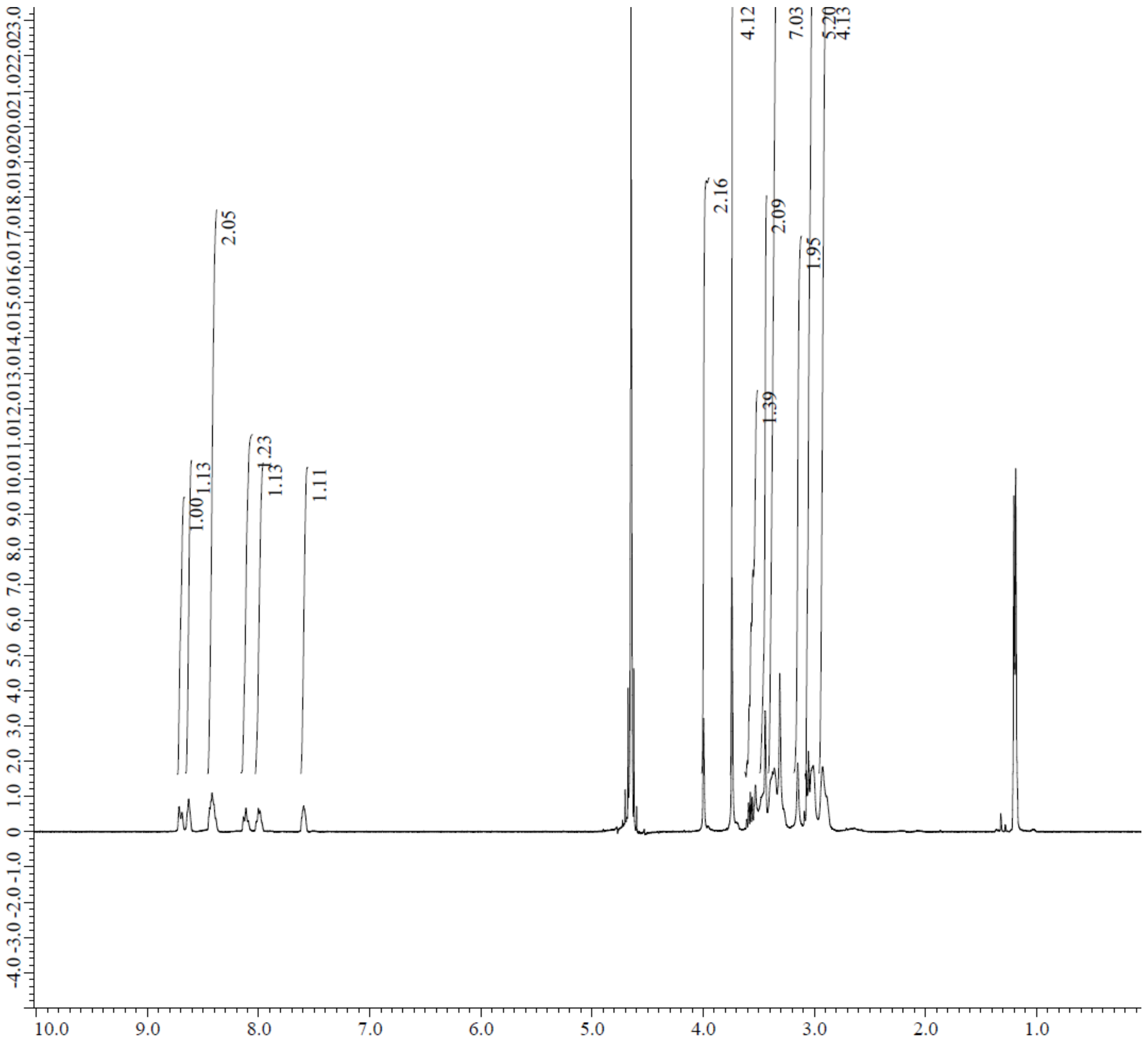

NOTA-3,6-di(2-pyridyl)-*s*-tetrazine, (**4**)

^{1}H NMR (400 MHz, D_2O)

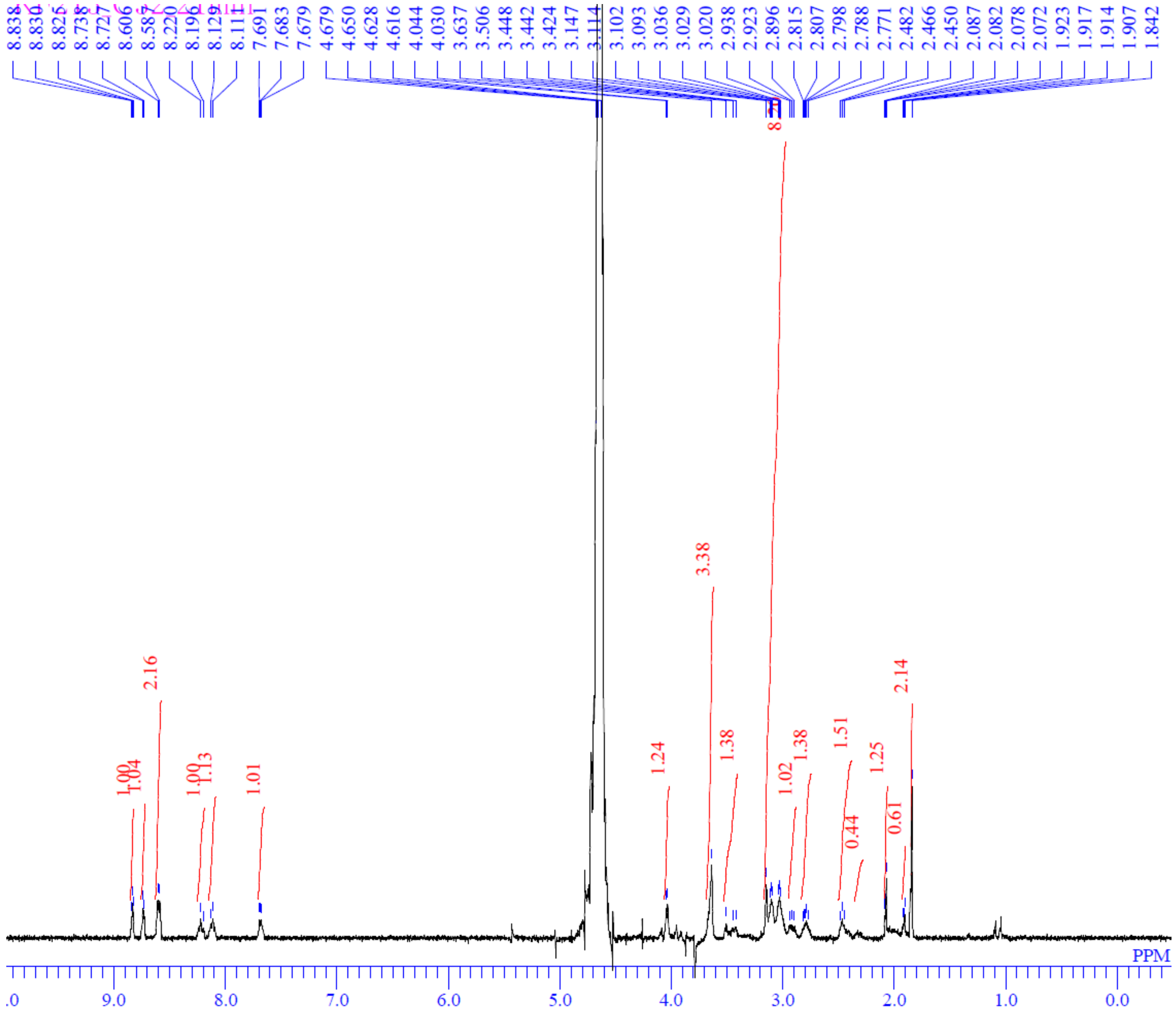

trans-cyclooctene-alcohol, (**C**)

^{1}H NMR (400 MHz, $CDCl_3$)

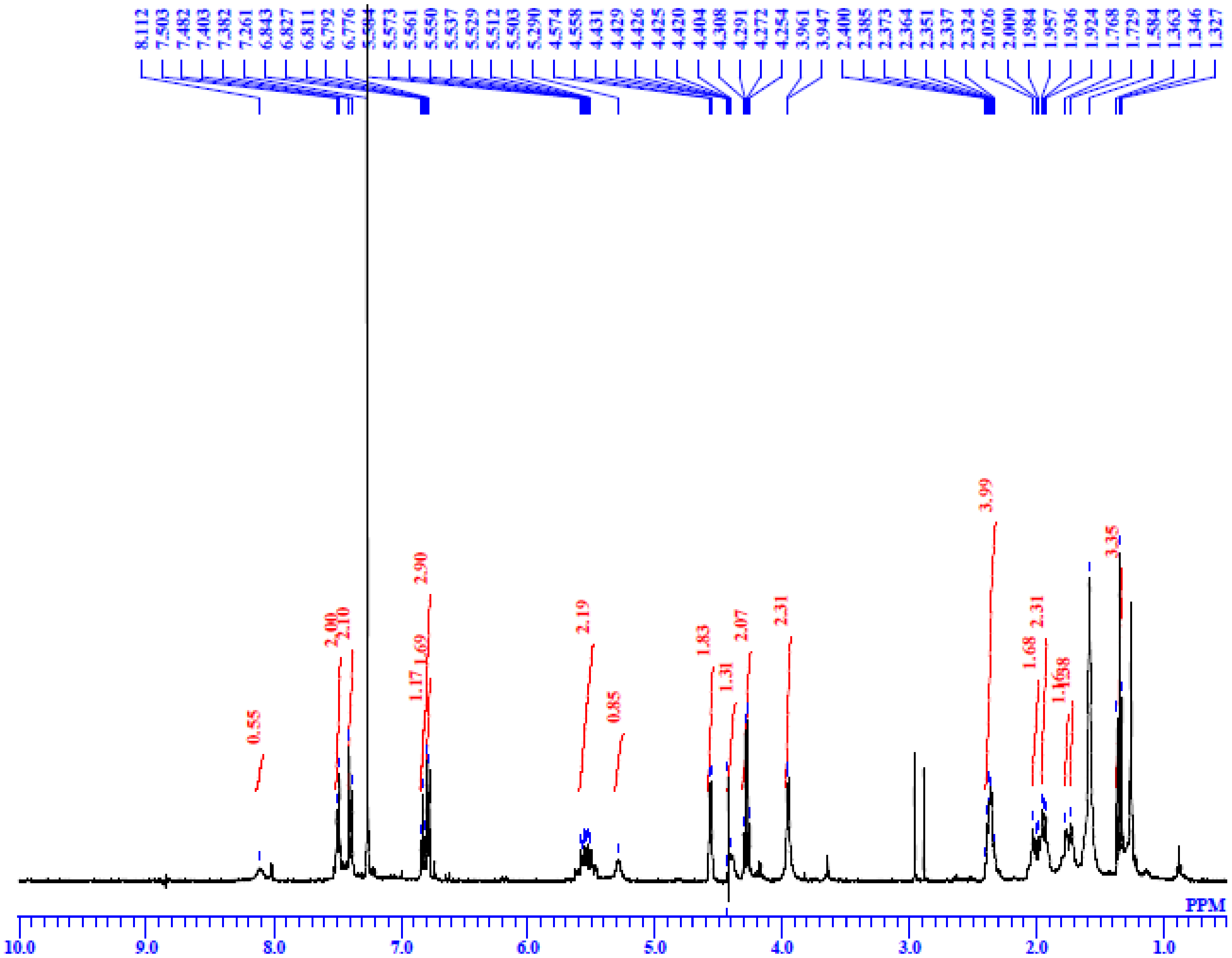

trans-cyclooctene-aldehyde, (**5**)

^{1}H NMR (400 MHz, $CDCl_3$)

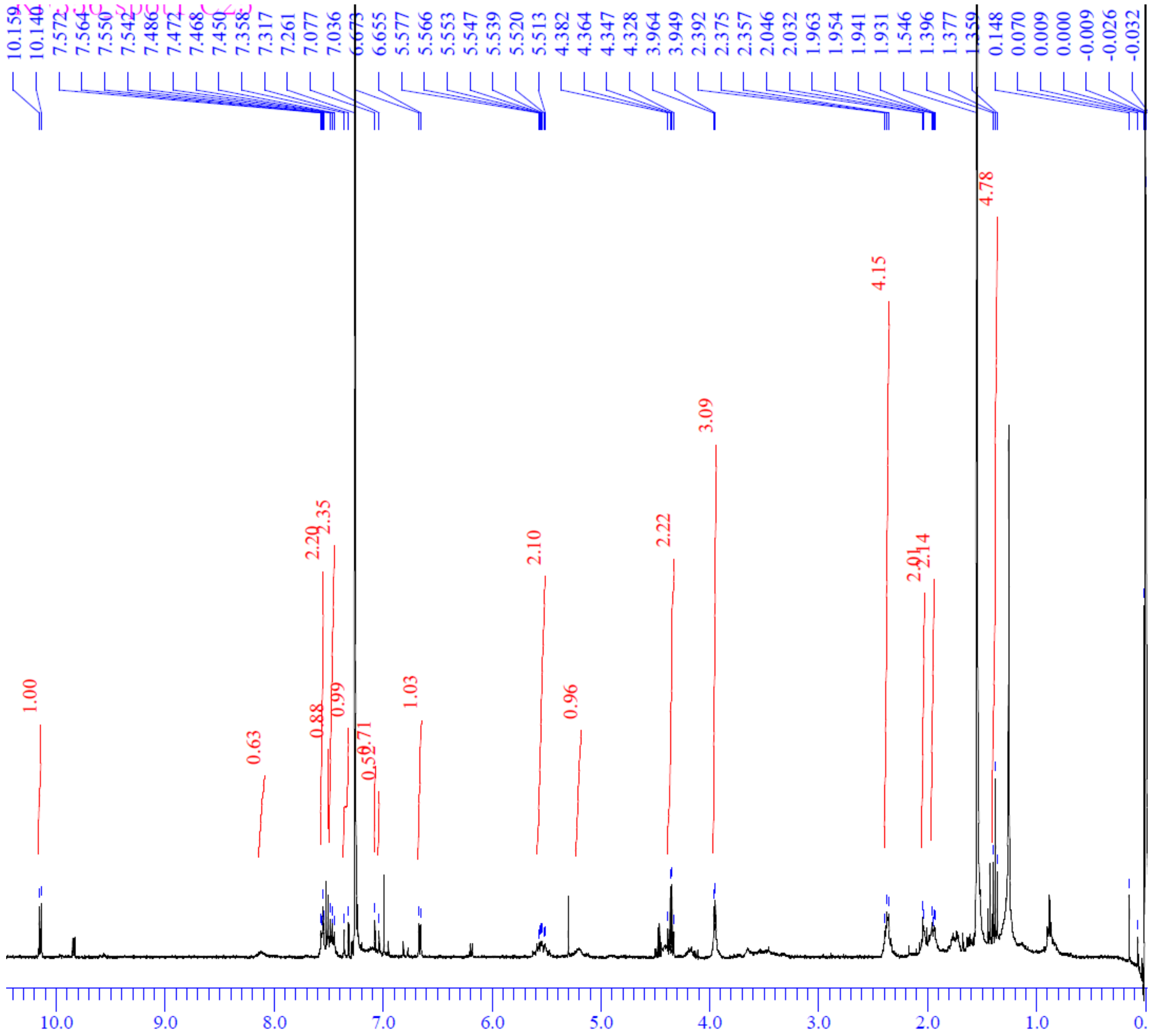

9. References

1. Blackman, M. L., Royzen, M. & Fox, J. M. *J. Am. Chem. Soc.* **2008**, *130*, 13518–13519.
